# Correction: Novel valdecoxib derivatives by ruthenium(ii)-promoted 1,3-dipolar cycloaddition of nitrile oxides with alkynes – synthesis and COX-2 inhibition activity

**DOI:** 10.1039/c8md90011f

**Published:** 2018-03-07

**Authors:** Silvia Roscales, Nicole Bechmann, Daniel Holger Weiss, Martin Köckerling, Jens Pietzsch, Torsten Kniess

**Affiliations:** a Department of Radiopharmaceutical and Chemical Biology , Helmholtz-Zentrum Dresden-Rossendorf , Institute of Radiopharmaceutical Cancer Research , Bautzner Landstraße 400 , 01328 Dresden , Germany . Email: t.kniess@hzdr.de; b Department of Inorganic Solid State Chemistry , Institute of Chemistry , University of Rostock , Albert Einstein Straße 3a , 18059 Rostock , Germany; c Department of Chemistry and Food Chemistry , Technische Universität Dresden , Bergstraße 66 , 01062 Dresden , Germany

## Abstract

Correction for ‘Novel valdecoxib derivatives by ruthenium(ii)-promoted 1,3-dipolar cycloaddition of nitrile oxides with alkynes – synthesis and COX-2 inhibition activity’ by Silvia Roscales *et al.*, *Med. Chem. Commun.*, 2018, DOI: 10.1039/c7md00575j.



## 

The authors regret that the structure of compound **1c** in Scheme 4 was not correct. The corrected structure is shown below.

**Scheme 4:**
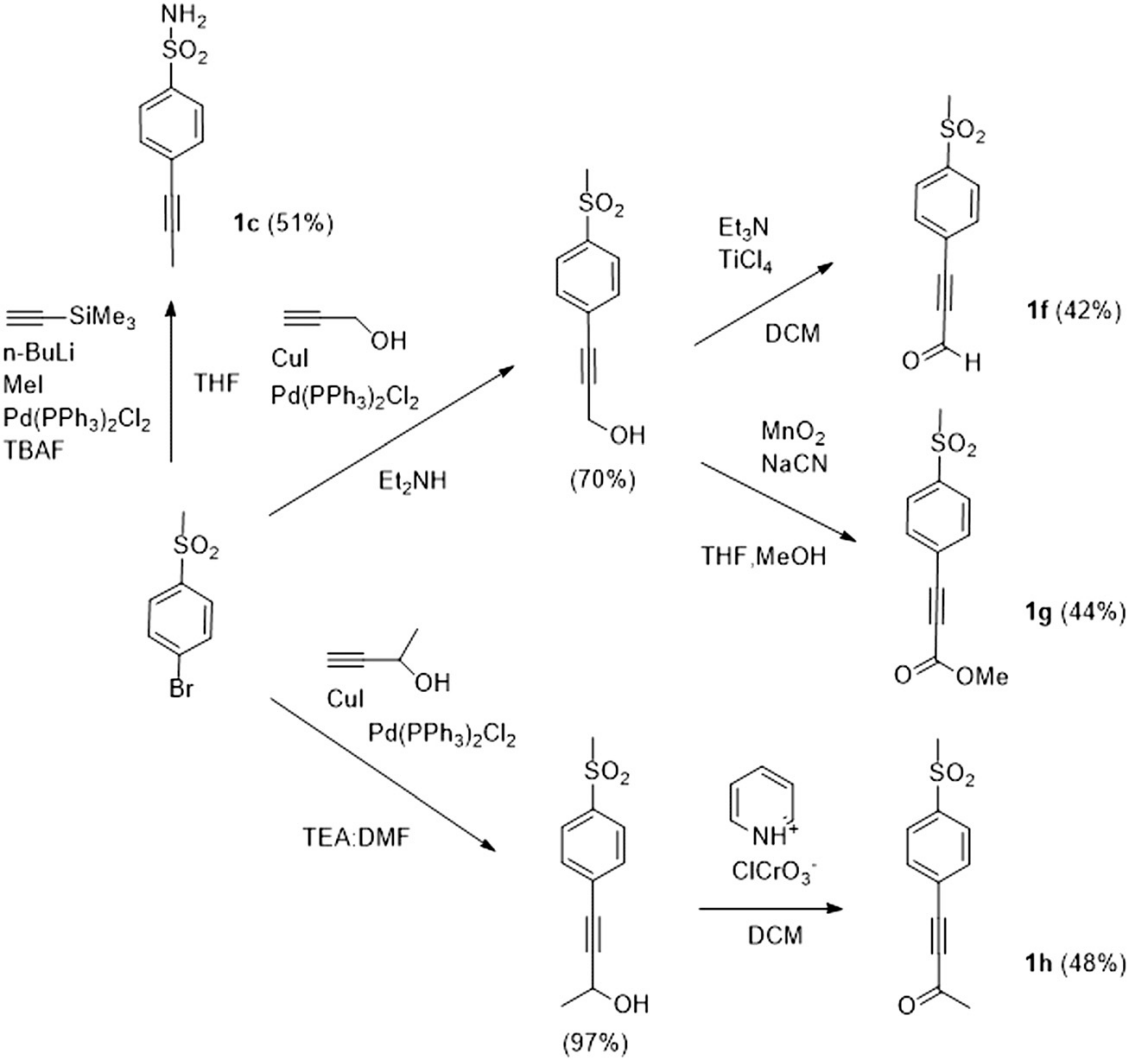


Also, the name of compound **1f** was not correct in the Electronic Supplementary Information file. 3-[4-(Methylsulfonyl)phenyl]propioaldehyde has been corrected to 3-[4-(methylsulfonyl)phenyl]prop-2-ynal and the corrected file has been uploaded to replace the original file.

The Royal Society of Chemistry apologises for these errors and any consequent inconvenience to authors and readers.

